# Epidermolysa bullosa in Danish Hereford calves is caused by a deletion in *LAMC2* gene

**DOI:** 10.1186/s12917-015-0334-8

**Published:** 2015-02-07

**Authors:** Leonardo Murgiano, Natalie Wiedemar, Vidhya Jagannathan, Louise K Isling, Cord Drögemüller, Jørgen S Agerholm

**Affiliations:** Institute of Genetics, Vetsuisse Faculty, University of Bern, Bremgartenstrasse 109a, CH-3001 Bern, Switzerland; Department of Veterinary Disease Biology, Section for Veterinary Pathology, Faculty of Health and Medical Sciences, University of Copenhagen, Ridebanevej 3, DK-1870 Frederiksberg C, Denmark; Department of Large Animal Sciences, Section for Veterinary Reproduction and Obstetrics, Faculty of Health and Medical Sciences, University of Copenhagen, Dyrlaegevej 68, DK-1870 Frederiksberg C, Denmark

**Keywords:** Cattle, Epidermolysis bullosa, Laminin gamma 2, Hereditary, Congenital, Skin

## Abstract

**Background:**

Heritable forms of epidermolysis bullosa (EB) constitute a heterogeneous group of skin disorders of genetic aetiology that are characterised by skin and mucous membrane blistering and ulceration in response to even minor trauma. Here we report the occurrence of EB in three Danish Hereford cattle from one herd.

**Results:**

Two of the animals were necropsied and showed oral mucosal blistering, skin ulcerations and partly loss of horn on the claws. Lesions were histologically characterized by subepidermal blisters and ulcers. Analysis of the family tree indicated that inbreeding and the transmission of a single recessive mutation from a common ancestor could be causative. We performed whole genome sequencing of one affected calf and searched all coding DNA variants. Thereby, we detected a homozygous 2.4 kb deletion encompassing the first exon of the *LAMC2* gene, encoding for laminin gamma 2 protein. This loss of function mutation completely removes the start codon of this gene and is therefore predicted to be completely disruptive. The deletion co-segregates with the EB phenotype in the family and absent in normal cattle of various breeds. Verifying the homozygous private variants present in candidate genes allowed us to quickly identify the causative mutation and contribute to the final diagnosis of junctional EB in Hereford cattle.

**Conclusions:**

Our investigation confirms the known role of laminin gamma 2 in EB aetiology and shows the importance of whole genome sequencing in the analysis of rare diseases in livestock.

**Electronic supplementary material:**

The online version of this article (doi:10.1186/s12917-015-0334-8) contains supplementary material, which is available to authorized users.

## Background

Heritable forms of epidermolysis bullosa (EB) constitute a heterogeneous group of skin disorders of genetic aetiology that are characterised by skin and mucous membrane blistering and ulceration in response to even minor trauma. EB is classified into four major types based on the level of blister formation in the dermo-epidermal interface, i.e. within the epidermis, basement membrane zone or uppermost dermis. In EB simplex, blisters develop within the epidermis, while for junctional and dystrophic EB cleavage occurs in the lamina lucida or below the lamina densa, respectively. The fourth major type, Kindler Syndrome, is characterized by blisters in the lamina lucida and below the lamina densa. In humans, many subtypes of which some have extracutaneous lesions have been identified [1-4].

In contrast to the situation in man, where more than 1000 mutations in at least 18 genes encoding structural proteins have been associated with EB and thousands of EB patients have been thoroughly diagnosed [3,4], rather few cases have been characterized to the molecular level in domestic animals. In cattle, EB simplex was associated with a mutation in *keratin 5* [5] and dystrophic EB was associated with *COL7A1* mutations in cattle [6] and dogs [7] while junctional EB has been diagnosed in sheep (*LAMC2* mutation) [8], horses (*LAMC2* and *LAMA3* mutations) [9-11], and dogs (*LAMA3* mutation) [12]. In addition to these, genetically uncharacterized EB cases in animals have been reported [13].

In addition to genetically characterised cases in cross-bred Holstein calves [5] and Rotes Höhenvieh cattle [6], sporadic [14-17] and multiple genetically linked cases within single herds have been reported [18-20]. In these cases, a presumptive diagnosis of EB was based on presence of congenital blistering of the skin and mucous membranes and histopathological detection of dermo-epidermal cleavage and in some cases supported by transmission electron microscopy (TEM) findings. Here we report the occurrence of EB in a Danish herd of Hereford cattle and its genetic characterization using positional cloning and whole genome sequencing.

## Methods

### Cases

The first case of EB, a female Hereford calf with a body weight of 30 kg, was born in March 2007 (case 1). The calf was euthanized four days old by intravenous injection of an overdose of pentobarbital sodium and submitted for necropsy. A second case of unregistered sex was stillborn in December 2007 (case 2). EB was diagnosed retrospectively based on the owner’s description of lesions as the calf was destroyed. The third case was a male Hereford calf with a body weight of 48 kg that died immediately after parturition in July 2009 (case 3) and was submitted for necropsy. The herd consisted of four breeding females in 2007. None of the parents of affected calves had signs of a blistering skin disorder. The study was performed according to Danish legislation and the cases published with the consent of the owner.

### Pathology

A complete necropsy was performed in both calves and specimens of skin and mucous membranes were sampled for histology. Samples were taken from within lesions, from the border between lesions and adjacent grossly normal tissue and from normal skin areas distant to lesions. Specimens were fixed in 10% neutral buffered formalin, processed by routine methods, embedded in paraffin, sectioned at 5 μm, and stained by haematoxylin and eosin. Selected sections were stained with periodic acid-Schiff (PAS).

### DNA samples and genotyping

Four-generation pedigrees of the cases were obtained from the Danish Cattle Database and analysed for inbreeding loops. Tissue samples were collected from the one of the available affected calves (case 1). In addition, blood samples were gathered from both parents of case 1 and the sire of case 3. DNA was extracted using standard methods. Genotyping of these animals was performed using the BovineHD BeadChip (illumina), including 777,961 evenly distributed single nucleotide polymorphisms (SNPs) and standard protocols as recommended by the manufacturer.

### Homozygosity mapping

PLINK software [21] was used to search for extended intervals of homozygosity with shared alleles as described previously [22]. Individuals and SNPs were selected using the commands --keep, and --extract while final files were generated through the --merge command. Homozygosity analysis was carried out on all cases using the commands --cow, --homozyg and --homozyg-group.

### Whole genome re-sequencing and searching for variants

A fragment library with a 300 bp insert size was prepared and collected in a single lane of Illumina HiSeq2500 paired-end reads (2 × 100 bp); the fastq files were created using Casava 1.8. We obtained a total of 487,657,379 paired-end reads, which were then mapped to the cow reference genome UMD3.1/bosTau6 and aligned using Burrows-Wheeler Aligner (BWA) version 0.5.9-r16 [23] with default settings. The mapping showed that 403,122,849 reads had unique mapping positions. The SAM file generated by BWA was then converted to BAM and the reads sorted by chromosome using samtools [24]. Polymerase chain reaction (PCR) duplicates were marked using Picard tools [25]. We used the Genome Analysis Tool Kit (GATK version 2.4.9, [26]) to perform local realignment and to produce a cleaned BAM file. The genome data have been made freely available under accession no. PRJEB7527 at the European Nucleotide Archive [27].

Search for variants was then made with the unified genotyper module of GATK. The variant data for each sample was obtained in variant call format (version 4.0) as raw calls for all samples and sites flagged using the variant filtration module of GATK. Variant filtration was performed following best practice documentation of GATK version 4. The snpEFF software [28] together with the UMD3.1/bosTau Ensembl annotation was used to predict the functional effects of detected variants. The Delly package was used to detect larger deletions in cleaned BAM files [29]. Delly uses variation in pair-end reads distance and orientation to find deletions. Structural variation software that are based on coverage and orientation are unable to detect variations larger than the insert size as read mapping software usually requires the library insert size as an argument for aligning within range. Hence, in order to avoid missing large inserts, deletions and false positives all detected variants in the candidate region were also manually inspected by visual control of the BAM file using IGV browser [30].

### Sanger sequencing

The *LAMC2* deletion was verified in the case and the available parents by re-sequencing of targeted PCR products using Sanger sequencing technology. PCR primers were designed using PRIMER3 [31]. PCR products were run on 0.8% agarose gel, 0.5 μg/ml ethidium bromide. PCR products were amplified using flanking primers for the *LAMC2* exon 1 deletion (F) GGCCTATAGAGAGTGGCATGA, (R) CAAATGAAGCCCTTTGAGGA and a second Reverse primer exclusive for the region deleted in the mutants TTCCTTCCCTCACCATCATC with AmpliTaqGold360Mastermix (Life Technologies) and the products directly sequenced using the PCR primers on an ABI 3730 capillary sequencer (Life Technologies) after treatment with exonuclease I (N.E.B.) and rAPid alkaline phosphatase (Roche). Sequence data were analyzed using Sequencher 5.1 (GeneCodes).

## Results and discussion

### Phenotypes

**Figure 1:**
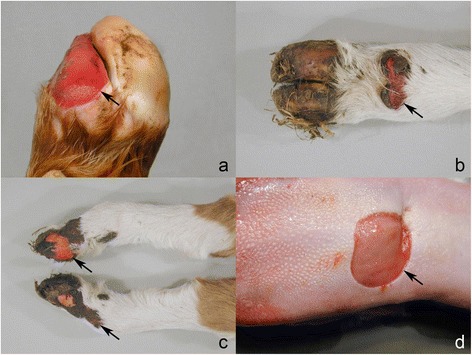
**Gross lesions in Hereford calves with epidermolysis bullosa. a)** Congenital absence of most of the hoof of a main digit (case 3); **b)** Loss of the horn of the dew claws with inflamed corium (case 1); **c)** Absence of part of the lateral aspect of the horn of the hind limb main digits exposing a hyperemic corium (case 1); **d)** Local absence of the lingual epithelium (case 1). The border of lesions is indicated by arrows.

Lesions in the skin were in principal similar in the two necropsied calves, but more widespread and severe in case 1 than in case 3 – probably reflecting the age difference (4 *vs.* 0 days of age). In case no. 3, skin lesions were restricted to bilateral absence of horn on the front limb dew claws and an area without horn affecting most of the hind limbs’ lateral main digit (Figure 1a). The exposed dermis was hyperaemic but without exudation. The border was sharply demarcated and the adjacent epidermis seemed normal. The few days old case (no. 1) had skin lesions in the distal parts of all limbs. The horn wall was absent on all dew claws exposing a hyperaemic corium covered by crusts (Figure 1b) and the horn on all main digits was defective. The horn wall was totally absent in one hind limb digit and partly absent in the others and the exposed corium showed intense hyperaemia (Figure 1c). The horn wall was loosened in both front limbs and separated from the corium in the coronet band with suppuration. Fistulas opened either in the coronet band or penetrated the sole. Skin ulcerations covered by crusts stretched proximally from the coronet band to the fetlock region in both hind limbs and were also found above the coronet band in the right front limb, in the left lateral metatarsal area and locally in the ventral part of the trunk.

Mucosal lesions were present in both necropsied cases. Case 3 had a large ulceration of the nasal plate with loosening of epidermis and stretching into the nostrils. In the oral cavity, both calves had extensive ulcerative lesions including a circular ulceration in the tongue around the anterior part of the torus (Figure 1d), and ulcerations in the palate, dental pad and adjacent area of the upper lip, gingiva, and cheeks.

### Histopathological changes

**Figure 2:**
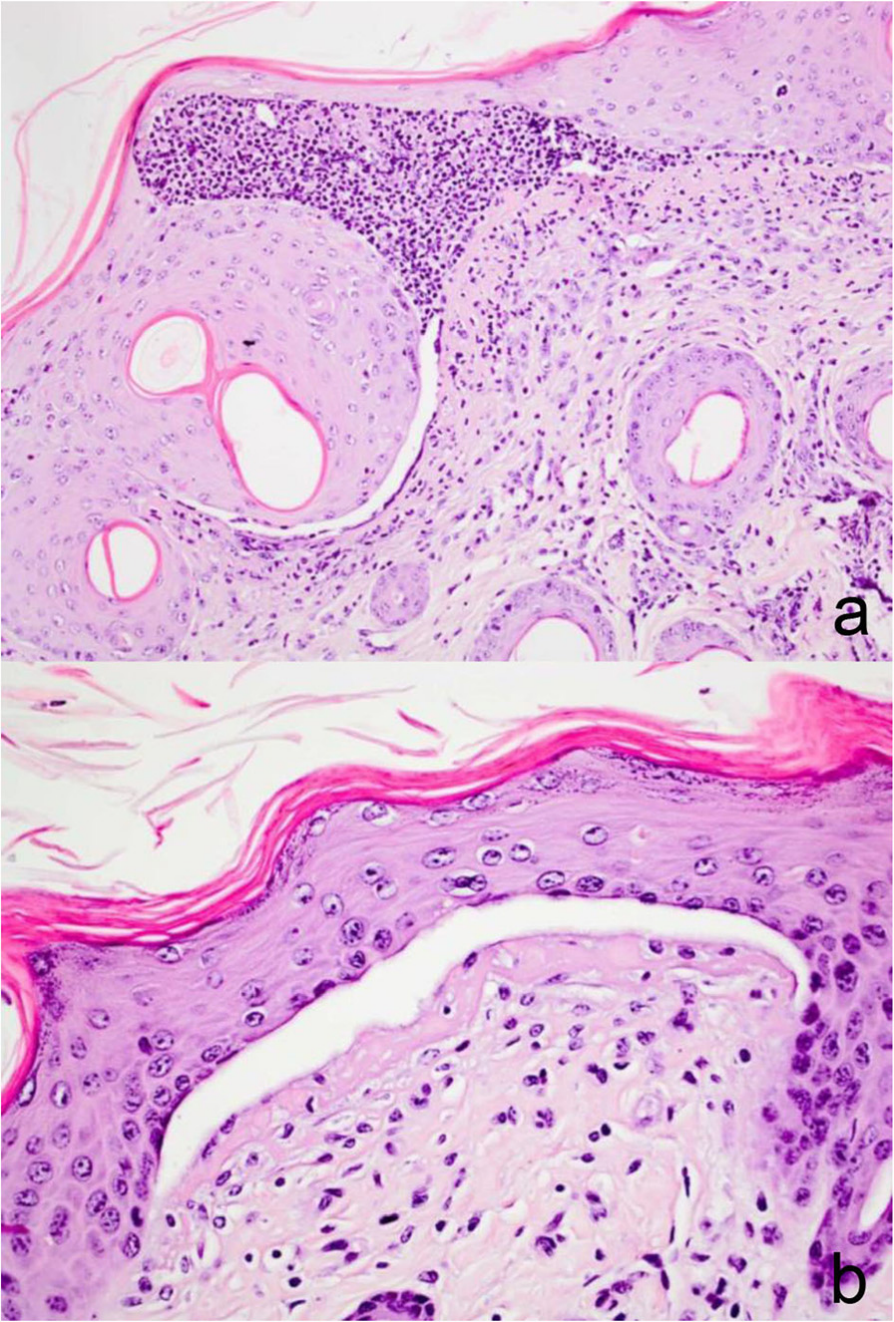
**Microscopic lesions in Hereford calves with epidermolysis bullosa. a)** Subepidermal blister with a purulent content and inflammation of the superficial dermis (case 1, hematoxylin and eosin, obj x 20); **b)** Subepidermal blister with just a few neutrophils, slight suppurative inflammation in the superficial dermis that is also covered by an eosinophilic material. The clear space may be artificial due to autolysis and tissue shrinkage during processing (case 1, hematoxylin and eosin, obj × 40).

Histopathological examination of the skin from the distal limbs of case 3, which died immediately after parturition, revealed an abrupt ulceration that was bordered by a hyperplastic epidermis. The skin proximal to the zone of epidermal hyperplasia was normal and without signs of dermoepidermal separation. The ulcerated area adjacent to the zone of epidermal hyperplasia showed acute mild suppurative inflammation, while more distant areas were dominated by an inflamed granulation tissue. Ulcerated areas where mostly without skin adnexa, i.e. glands and hair, although isolated hairs were rarely seen. Skin lesions of case 1 were dominated by ulcerations with superficial dermal necrosis, debris, and profound suppurative inflammation. The epidermis adjacent to the ulcerative area was necrotic and separated from the dermis and the dermis had diffuse suppurative inflammation. This zone continued into areas with subepidermal blisters with a purulent content (Figure 2a) and in more distant areas by subepidermal blisters with just a few neutrophils and decreasing degrees of dermal inflammation (Figure 2b). Remnants of skin adnexa were present in the ulcerated areas.

Lesions of the dental pad and upper lip of case 3 were characterised by an abrupt transition from normal epithelium to an ulcer with peripheral acute inflammation and distant granulation tissue formation with sparse inflammation. Beneath ulcerated lesions, profoundly located merocrine sweat glands were present. In case 1, dental pad and nasal plate lesions were characterised by severe fibrino-necrotising and suppurative inflammation of the denuded dermis and bordered by areas with subepidermal blisters. In the tongue, lesions corresponded to those observed in the dental pad of cases 1 and 3, respectively. PAS staining revealed a basement membrane apparently located at the bottom of some blisters, while a distinct basement membrane was not present in others. An EB type/subtype was not established as appropriate materials were not available. Tissues were autolysed due to prolonged time between the death of the calves and necropsy and cryopreserved specimens for immunofluorescence antigen mapping were not sampled. Typing of EB is severely compromised if optimal specimens for immunofluorescence antigen mapping are not sampled and diagnostic based on formalin fixed tissues and suboptimal TEM examinations may be misleading [1,32] in microscopic typing of EB.

The calves had a severe congenital blistering disorder affecting the skin and mucous membranes, and the claws and dewclaws had either total or partial loss of horn. Histologically, blisters were present in the areas of the skin affected in EB although the precise localization of the spitting plane could not be determined. In combination, these findings are consistent with EB and the cases share many features with established or suspected cases of EB in cattle [6,16-20].

### Pedigree analysis and mapping

**Figure 3:**
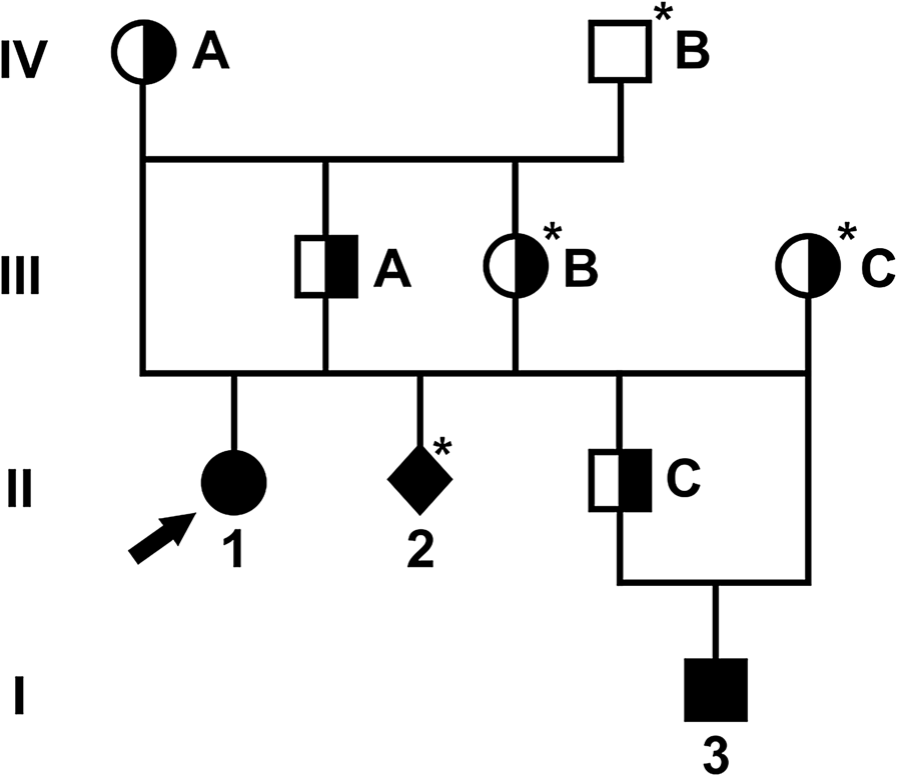
**Genealogical diagram showing three Hereford calves affected by epidermolysis bullosa and their parents.** Males are represented by squares, females by circles, animals of unknown sex are shown rhomboid. Affected animals are shown with fully black symbols and carriers with a half-filled symbol. Note that four of the animals were not genotyped since the DNA was not available (IV/B, II/2, III/B and III/C, indicated with an asterisk). Individuals III/B and III/C are shown with half-filled symbols since they are obligate carriers. The sequenced case is indicated by an arrow.

The close familiar relationship between cases strongly indicated a genetic aetiology; we therefore decided to analyse the pedigree data to infer an inheritance mechanism. Pedigree data were not complete, but the available information regarding ancestors allowed us to draw a genealogical diagram (Figure 3). Analysis of the diagram indicated that inbreeding and the transmission of a recessive mutant allele from a common ancestor; either cow IV/A or Sire IV/B (Figure 3) could be the founder or the distributor of the responsible mutation. These animals had been mated and produced a son (III/A), who was bred to his own mother (IV/A). This inbreeding loop produced case 1; III/A was also bred to his sister (III/B) and produced case 2 and got a son (II/C) with a cow of unknown descent (III/C). The son II/C was mated to his mother (III/B), who gave birth to case 3. III/C and IV/A could share a common ancestor - presence of other common ancestors could not be excluded due to incomplete pedigree data. These data suggested a monogenic recessive inheritance mechanism; therefore, we hypothesized a simple Mendelian recessive inheritance was the most likely explanation for the condition. We initiated a positional cloning study to unravel the underlying genetics. We assumed that the affected calves were expected to be identical by descent (IBD) for the causative mutation and flanking chromosomal segments. We initially genotyped 777,961 evenly spaced SNPs one family trio (case 1 plus its parents). We searched for extended regions of homozygosity and compared the homozygous region between the case and the parents. Interestingly, in the genotyped affected animal about 37% of the genome is homozygous as expected in a consanguineous son-mother mating (Figure 3). We excluded any homozygous regions already present in the parents. Thereby, we found 40 genome regions greater than 1 Mb that fulfilled these criteria (Additional file 1).

**Figure 4:**
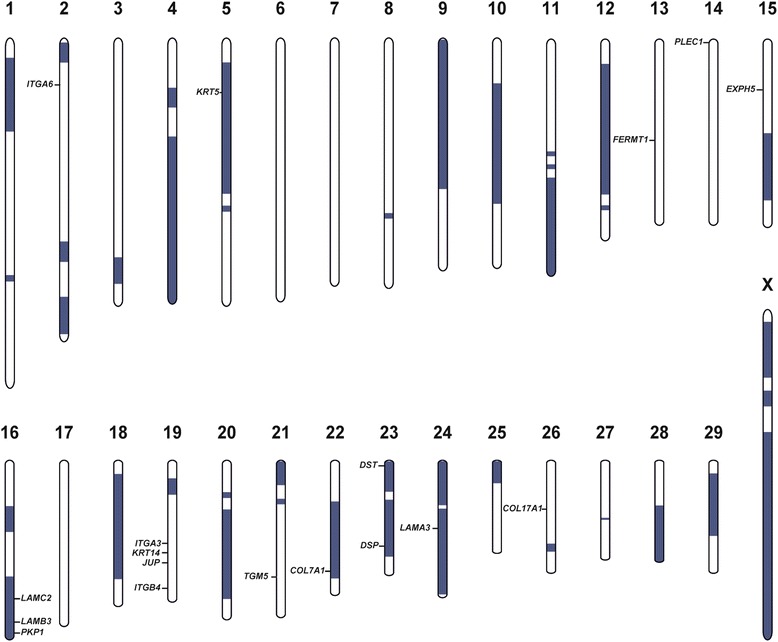
**Homozygosity mapping and position of known epidermolysis bullosa candidate genes in the cattle genome.** Extended segments of private homozygosity in case 1 are shown in blue. Note, that 8 out of 18 EB genes including *LAMC2* are located in homozygous regions.

In the light of the few reports of EB causing mutations in livestock we hypothesized that a causative variant might affect one of the known EB candidate genes. Eight out of 18 EB candidates mapped in the identified homozygous regions (Figure 4). We sequenced the whole genome of case 1 at 17.5× coverage of the genome and proceeded to screen the candidate genes present in the mapped homozygous intervals for possible variants. This allowed us to identify 118,014 single nucleotides and short insertion/deletion variants within the whole exome. From this point, we decided to use a candidate gene based approach. We carefully checked for all the variants present in the coding sequence of the 8 remaining EB candidate genes, which were located in the previously identified homozygous candidate regions (Figure 4). We found no homozygous private variant in all EB genes in the sequenced affected animal after comparison with available data of 40 sequenced control cattle genomes (Additional file 2), which were sequenced in the course of other ongoing projects in our group (variants exclusive of the sequenced animal after this filtering step are reported in Additional file 3).

**Figure 5:**
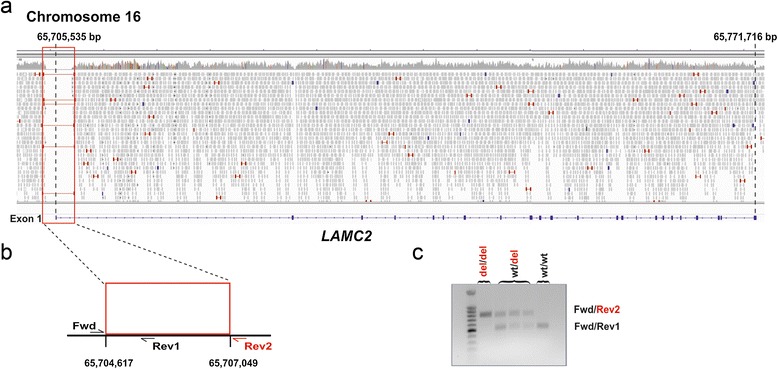
***LAMC2*** **deletion associated with epidermolysis bullosa in Hereford cattle. a)** Screenshot of the next generation sequence reads mapped against the reference sequencing and visualized with Integrative Genome Viewer. Note the 2433 bp deletion including the first exon of *LAMC2*. **b)** We used a common forward primer in combination with a reverse primer specific for the deleted region (Rev1) and a reverse primer located immediately downstream of the deletion (Rev2) for PCR based deletion genotyping. Detected genotypes of the *LAMC2* deletion. **c)** The diagnostic PCR performed on genomic DNA allows genotype differentiation. The gel picture shows the affected calf (del/del), three heterozygous parents (wt/del), and a normal control (wt/wt).

Using these controls genomes we went on searching for larger deletions and found a total of 349 private deletions occurring only in the genome of the affected calf (Additional file 4). Interestingly, among these deletions the only variant detected overlapping with an annotated coding region found was a 2,433 bp deletion on chromosome 16 (g.65,704,617_65,707,049del) affecting an EB candidate gene. This homozygous deletion encompasses the region more than 900 bp upstream and 1.1 kb downstream of the first exon of the annotated transcript *ENSBTAT00000061289.* This annotated bovine transcript corresponds to the human *LAMC2* gene encoding the laminin gamma 2 protein. The variant causes the complete deletion of the entire *LAMC2* exon 1 containing the start codon and the first 79 coding bases of the transcript (Figure 5). We genotyped the available family members (dam of case 1, IV/A; sire of case 1, III/A; sire of case 3, II/C) and found the mutation present in heterozygosity in these animals (Figure 5). This confirms the assumed recessive inheritance of the EB mutation within this cattle family. The mutant allele was absent in normal controls. A homozygous mutation completely removing the start codon of the evolutionary conserved *LAMC2* wildtype transcript is highly likely disruptive and almost certainly negates completely the presence of a functional LAMC2 protein. Alternatively, the possible usage of a second start codon located approximately 900 nucleotides further downstream is predicted to lead to a truncated protein lacking about 25% of the wildtype *LAMC2* including three conserved domains. Therefore we speculate that this mutant protein will, if really expressed, probably not compensate the physiological function of the wild type protein. The *LAMC2* loss of function mutation affects a well-known candidate gene associated with junctional EB in humans [33-40] and domestic animals like sheep and horse [8,9]. For this reason, we concluded that the observed EB type in this cattle family was caused by the detected *LAMC2* deletion.

#### Clinical and molecular characterization of EB

Diagnostic of EB in animals, including classification in major types, has been done in several studies based on histology and TEM. However, routine histological processing of skin is not recommended by human pathologists for EB diagnostic due to the difficulties in distinguishing at the light microscopy level between several types of EB [4]. Furthermore, correct sampling and processing of specimens is crucial to avoid artifactual blistering. Also fresh blisters should be induced by gently rubbing the skin rather that sampling older blisters as this may lead to a wrong diagnosis [41]. TEM examination has been used in several studies on EB in animals but require samples without autolysis. This may be difficult to achieve as animals often die or are euthanized on the farm by humane grounds before appropriate sampling is possible as for the cases reported here. Furthermore, processing of skin biopsy specimens for TEM and interpretation of EB ultrastructural changes require extensive experience and expertise that is available only in a very few recommended reference laboratories worldwide. Otherwise findings may be quite misleading and should be interpreted with caution [4]. Immunofluorescence mapping for antigens associated with EB in cryopreserved skin specimens taken from fresh spontaneous or traction-force induced blisters is the recommended diagnostic method, especially if samples are shipped to a reference laboratory [4]. The recommendations by experts in EB in man [4] shows that there are many pitfalls in non-molecular diagnostic of EB that are obstacles in veterinary diagnostic, especially due to suboptimal materials and lack of experience with the highly specialised EB diagnostic.

Congenital localised absence of skin (CLAS) and mucosal epithelium was apparent in the calf that died immediately after parturition (case 3). CLAS has been reported as a manifestation of EB in man, e.g. Bart’s Syndrome [42-44] and although CLAS and mucosal epithelium defects may be due inadequate development, CLAS in association with EB merely reflects intrauterine loss of tissue due to foetal development of EB [43].

Congenital blistering disorders in the mucous membranes, muzzle and skin, especially the distal part of the limbs, have been reported in cattle through decades and usually referred to as “epitheliogenesis imperfecta” (EI) as proposed by Hadley [45]. Many of these cases have occurred in familial patterns associated with inbreeding and likely transmission of an autosomal recessive mutant allele from a founder animal [44-46] and many cattle breeds have been affected [15,45-51]. However, EI probably comprises of two distinct entities, namely congenital cutaneous aplasia (CCA) and EB and taking the current knowledge on the genetic background and variation even within subtypes of EB in man into consideration, the reported cases are likely to have different etiopathogeneses. Investigation of CCA has demonstrated that this is not a blistering disease and it is striking that cases resembling EI (except CCA cases) have turned out to be EB if investigated to the molecular level [5,6,9,10,52]. Furthermore, it is worth remembering that EI was introduced to veterinary medicine based on gross lesions only [44]. It is therefore proposed that EI is no longer used as a disease entity but replaced by either CCA or EB as also suggested by others [6,53].

EB is a rare disease known in man and many animal species. However, in livestock subtypes with recessive inheritance have the potential to become of significance if the defective allele is present in important sires used for artificial breeding through several years as seem for other genetic diseases in cattle [54-56]. It is therefore important that cases are diagnosed to subtype level when the first cases are recognised to enable rapid development of genetic test that allows screening of the sire population. However, subtyping of EB is a task for clinicians and pathologists, but it starts with clinical examination and sampling and processing of adequate materials for microscopic and genomic analysis [41]. The quick identification of an highly disruptive mutation in a known candidate gene allowed us to unambiguously identify and classify the EB cases in Hereford and thus (I) allow for possible screening of the mutation in the Danish Hereford population as preventive measure (II) giving a new animal model for EB caused by a mutation in laminin gamma genes.

## Conclusions

The study reports for the first time the occurrence of EB in Hereford cattle. Our investigation confirms the role of a recessively inherited *LAMC2* loss of function mutation in EB aetiology. Next-generation sequencing offers a powerful tool for understanding the genetic background of rare diseases in domestic animals with an available reference genome sequence. Verifying the causative mutation allowed us to confirm to the diagnosis of EB and allows breeders to eliminate this genetic defect from the population.

## Additional files

Additional file 1:
**Private regions of homozygosity detected in epidermolysis bullosa case 1.** Only homozygous regions exclusive for the case are shown. Additional file 2:
**List of all the control cattle genomes and their breed.**
Additional file 3:
**List of all the private homozygous variants of the sequenced epidermolysis bullosa case 1.**
Additional file 4:
**Private larger deletions detected in epidermolysis bullosa case 1.** Deletion in encompassing *LAMC2* exon 1 is highlighted.

## Abbreviations

Bp: Base pairs
CCA: Congenital cutaneous aplasia
CLAS: Congenital localised absence of skin
EB: Epidermolysis bullosa
EI: Epitheliogenesis imperfect
kb: Kilo bytes
PAS: Periodic acid-Schiff
PCR: Polymerase chain reaction
SNP: Single nucleotide polymorphism
TEM: Transmission electron microscopy
