# Supplementary material for: Epidermolysa bullosa in Danish Hereford calves is caused by a deletion in LAMC2 gene
Source: BMC Vet Res. 2015 Feb 7;11:23. doi: 10.1186/s12917-015-0334-8 (PMC4328060; doi:10.1186/s12917-015-0334-8)
Supplement: Additional file 1: — Private regions of homozygosity detected in epidermolysis bullosa case 1. Only homozygous regions exclusive for the case are shown. [file 12917_2015_334_MOESM1_ESM.docx]

**Additional file 1. Private regions of homozygosity detected in epidermolysis bullosa case 1.** Only homozygous regions exclusive for the case are shown. CHR: Chromosome. SNP1, SNP2: name of the flanking SNPs. BP1, BP2: base pair position of the SNP1 and SNP2, respectively. Size (Kb): size of the region in kilobases.

| **CHR** | **SNP1** | **SNP2** | **Position 1** | **Position 2** | **Size (bp)** |
| --- | --- | --- | --- | --- | --- |
| *1* | BovineHD0100002487 | BovineHD0100011704 | 7766208 | 41031010 | 33264802 |
| *1* | BovineHD0100031155 | BovineHD0100031413 | 109926178 | 111119754 | 1193576 |
| *2* | BovineHD0200000001 | BovineHD0200002736 | 6889 | 9614468 | 9607579 |
| *2* | BovineHD0200026583 | BovineHD0200031987 | 93162321 | 110980146 | 17817825 |
| *2* | BovineHD0200034930 | BovineHD0200040109 | 120388252 | 136908437 | 16520185 |
| *3* | BovineHD0300028781 | BovineHD0300032122 | 100318836 | 111703942 | 11385106 |
| *4* | BovineHD0400006560 | BovineHD0400007268 | 22165198 | 24797186 | 2631988 |
| *4* | BovineHD0400007274 | BovineHD0400008567 | 24815450 | 120786530 | 95971080 |
| *5* | BovineHD0500000859 | BovineHD0500001201 | 3378428 | 4604237 | 1225809 |
| *5* | BovineHD0500024302 | ARS-BFGL-NGS-27744 | 85814584 | 102152540 | 16337956 |
| *8* | BovineHD0800024061 | BovineHD0800024333 | 80678614 | 81928187 | 1249573 |
| *9* | BovineHD0900031325 | BovineHD0900018872 | 10049 | 68204475 | 68194426 |
| *10* | BovineHD1000006986 | BovineHD1000031198 | 21513571 | 74003650 | 52490079 |
| *11* | BovineHD1100014853 | BovineHD1100015326 | 50423056 | 52294718 | 1871662 |
| *11* | BovineHD1100016407 | BovineHD1100016743 | 56290149 | 58128140 | 1837991 |
| *11* | Hapmap40852-BTA-99129 | BovineHD1100031142 | 63209412 | 107083925 | 43874513 |
| *12* | BTB-00486553 | BovineHD1200019361 | 11414743 | 70426762 | 59012019 |
| *12* | BovineHD1200021338 | BovineHD1200022309 | 76074162 | 78371110 | 2296948 |
| *15* | BovineHD1500000003 | BovineHD1500013742 | 43737 | 64793885 | 64750148 |
| *16* | BovineHD1600005838 | BovineHD4100012444 | 20984708 | 32056735 | 11072027 |
| *16* | BovineHD1600014972 | BovineHD1600024109 | 53936564 | 81720984 | 27784420 |
| *18* | BovineHD1800002181 | BovineHD1800015950 | 6414067 | 54362709 | 47948642 |
| *19* | BovineHD1900000002 | BovineHD1900004321 | 81083 | 15884314 | 15803231 |
| *20* | BovineHD2000004439 | BovineHD2000004840 | 14085613 | 16095658 | 2010045 |
| *20* | BovineHD2000006852 | BovineHD2000018180 | 22749631 | 63920603 | 41170972 |
| *21* | BovineHD2100000001 | BovineHD2100002688 | 5191 | 10986359 | 10981168 |
| *21* | BovineHD2100004981 | BovineHD2100005492 | 17736062 | 19343343 | 1607281 |
| *22* | BovineHD2200005537 | BovineHD2200015165 | 19073643 | 53445029 | 34371386 |
| *23* | BovineHD2300000001 | BovineHD2300003509 | 10121 | 14074758 | 14064637 |
| *23* | BovineHD2300015387 | BovineHD2300007048 | 17914012 | 44378079 | 26464067 |
| *24* | BovineHD2400000002 | BTB-01448403 | 54398 | 54603246 | 54548848 |
| *24* | BovineHD2400015957 | ARS-BFGL-NGS-83184 | 55879666 | 62685898 | 6806232 |
| *25* | BovineHD2500000005 | BovineHD2500002930 | 25945 | 10864756 | 10838811 |
| *26* | BovineHD2600010584 | BovineHD2600011597 | 38509126 | 41919030 | 3409904 |
| *27* | BovineHD2700006565 | BovineHD2700007713 | 23371908 | 27483274 | 4111366 |
| *28* | BovineHD2800003586 | BovineHD2800013502 | 12386658 | 46248750 | 33862092 |
| *29* | BovineHD2900001703 | BovineHD2900015459 | 6191963 | 34505551 | 28313588 |
| *30* | BovineHD3000001697 | BovineHD3000006118 | 4853972 | 30527830 | 25673858 |
| *30* | BovineHD3000046373 | BovineHD3000012124 | 36587401 | 43436412 | 6849011 |
| *30* | BovineHD3000016267 | BovineHD3000045078 | 55585487 | 148820237 | 93234750 |
