# Supplementary material for: Epidermolysa bullosa in Danish Hereford calves is caused by a deletion in LAMC2 gene
Source: BMC Vet Res. 2015 Feb 7;11:23. doi: 10.1186/s12917-015-0334-8 (PMC4328060; doi:10.1186/s12917-015-0334-8)
Supplement: Additional file 2: — List of all the control cattle genomes and their breed. [file 12917_2015_334_MOESM2_ESM.docx]

**Additional file 2. List of all the control cattle genomes and their breed**

| Sample Name | Breed |
| --- | --- |
| Control 1 | Simmental |
| Control 2 | Holstein |
| Control 3 | Simmental |
| Control 4 | Simmental |
| Control 5 | Scotish Highland Cattle |
| Control 6 | Brown Swiss |
| Control 7 | Brown Swiss |
| Control 8 | Pezzata Rossa Italiana |
| Control 9 | Holstein x Simmental |
| Control 10 | Holstein |
| Control 11 | Holstein |
| Control 12 | Charolais |
| Control 13 | Hereford |
| Control 14 | Scotish Highland Cattle |
| Control 15 | Eringer |
| Control 16 | Brown Swiss |
| Control 17 | Holstein |
| Control 18 | Holstein |
| Control 19 | Brown Swiss |
| Control 20 | Belted Galloway |
| Control 21 | Eringer |
| Control 22 | Romagnola |
| Control 23 | Holstein |
| Control 24 | Holstein |
| Control 25 | Limousin x Holstein |
| Control 26 | Holstein |
| Control 27 | Limousin |
| Control 28 | Tyrolean Grey Cattle |
| Control 29 | Belted Galloway |
| Control 30 | Piemontese x Normande |
| Control 31 | Simmental |
| Control 32 | Simmental |
| Control 33 | Simmental |
| Control 34 | Holstein |
| Control 35 | Romagnola |
| Control 36 | Holstein |
| Control 37 | Holstein |
| Control 38 | Tyrolean Grey Cattle |
| Control 39 | Holstein |
| Control 40 | Holstein |
