# Supplementary material for: Epidermolysa bullosa in Danish Hereford calves is caused by a deletion in LAMC2 gene
Source: BMC Vet Res. 2015 Feb 7;11:23. doi: 10.1186/s12917-015-0334-8 (PMC4328060; doi:10.1186/s12917-015-0334-8)
Supplement: Additional file 3: — List of all the private homozygous variants of the sequenced epidermolysis bullosa case 1. [file 12917_2015_334_MOESM3_ESM.docx]

**Additional file 3**. **List of all the private homozygous variants of the sequenced epidermolysis bullosa case 1.** CHR: Chromosome; POS: Position; REF: base on the reference sequence; ALT: polymorphism; Type: classification of the mutation; AA mod: possible aminoacid modification; Gene: annotated gene on the sequence.

| **CHR** | **POS** | **REF** | **ALT** | **Type** | **AA mod** |  | **Gene** |  |  |
| --- | --- | --- | --- | --- | --- | --- | --- | --- | --- |
| **2** | 99740623 | A | G | INTRON |  | | ENSBTAG00000012647 | | |
|  |  | A | G | SPLICE_SITE_REGION |  | | ENSBTAG00000012647 | | |
| **2** | 103925001 | C | T | SYNONYMOUS_CODING | T703 | | B8Y9S9_BOVIN | | |
| **2** | 106635911 | G | A | NON_SYNONYMOUS_CODING | P1593L | | A1A4J4_BOVIN | | |
| **2** | 107376082 | G | A | NON_SYNONYMOUS_CODING | V790I | | STK36 | | |
| **2** | 107412318 | C | T | NON_SYNONYMOUS_CODING | A94V | | TTLL4 | | |
| **2** | 108137506 | G | A | NON_SYNONYMOUS_CODING | V2039I | | SPEG | | |
| **2** | 108144268 | C | T | NON_SYNONYMOUS_CODING | P2929S | | SPEG | | |
| **2** | 108145426 | G | A | NON_SYNONYMOUS_CODING | R3130Q | | SPEG | | |
| **2** | 108153456 | A | G | START_GAINED |  | | GMPPA | | |
|  |  | A | G | UTR_5_PRIME |  | | GMPPA | | |
| **2** | 108222733 | G | A | SYNONYMOUS_CODING | H480 | | Q0VC42_BOVIN | | |
|  |  | G | A | SYNONYMOUS_CODING | H67 | | Q0VC42_BOVIN | | |
|  |  | G | A | UPSTREAM |  | | INHA_BOVIN | | |
| **2** | 125829925 | G | C | SYNONYMOUS_CODING | T59 | | ENSBTAG00000009937 | | |
|  |  | G | C | SYNONYMOUS_CODING | T60 | | ENSBTAG00000009937 | | |
| **2** | 125829940 | C | T | SYNONYMOUS_CODING | D64 | | ENSBTAG00000009937 | | |
| **2** | 125829940 | C | T | SYNONYMOUS_CODING | D65 | | ENSBTAG00000009937 | | |
| **2** | 126353148 | C | T | SYNONYMOUS_CODING | D995 | | AHDC1 | | |
| **2** | 126354213 | C | T | SYNONYMOUS_CODING | D1350 | | AHDC1 | | |
| **2** | 130781939 | C | T | DOWNSTREAM |  | | Q1RMH5_BOVIN | | |
|  |  | C | T | NON_SYNONYMOUS_CODING | G103E | | ENSBTAG00000011195 | | |
| **2** | 130836514 | C | T | INTRON |  | | EPHA8 | | |
|  |  | C | T | SPLICE_SITE_REGION |  | | EPHA8 | | |
| **2** | 130937573 | C | T | SYNONYMOUS_CODING | S140 | | ZBTB40 | | |
| **2** | 130937798 | G | A | SYNONYMOUS_CODING | V65 | | ZBTB40 | | |
| **2** | 130937974 | T | C | NON_SYNONYMOUS_CODING | S7G | | ZBTB40 | | |
| **2** | 131315470 | C | A | SYNONYMOUS_CODING | G214 | | WNT4 | | |
| **3** | 101938982 | C | T | UPSTREAM |  | | TMM53_BOVIN | | |
|  |  | C | T | UTR_5_PRIME |  | | CA228_BOVIN | | |
| **3** | 103596650 | T | C | NON_SYNONYMOUS_CODING | V136A | | ENSBTAG00000023748 | | |
| **3** | 103608553 | T | C | NON_SYNONYMOUS_CODING | F225L | | ENSBTAG00000037770 | | |
| **3** | 104118247 | G | A | NON_SYNONYMOUS_CODING | A177V | | YBX1 | | |
| **4** | 44862290 | A | G | NON_SYNONYMOUS_CODING | F23S | | SLC26A5 | | |
| **4** | 45682227 | C | G | UTR_5_PRIME |  | | A5PJS5_BOVIN | | |
| **4** | 49275001 | C | T | SYNONYMOUS_CODING | V1368 | | LAMB1 | | |
| **4** | 49553672 | C | T | NON_SYNONYMOUS_CODING | G1065D | | NRCAM | | |
| **4** | 55880614 | G | A | NON_SYNONYMOUS_CODING | A344V | | IFRD1 | | |
| **4** | 66612471 | T | C | UTR_3_PRIME |  | | CG041_BOVIN | | |
| **4** | 66612887 | G | C | UTR_3_PRIME |  | | CG041_BOVIN | | |
| **4** | 66613537 | T | C | UTR_3_PRIME |  | | CG041_BOVIN | | |
| **4** | 66613547 | TAC | T | UTR_3_PRIME |  | | CG041_BOVIN | | |
| **4** | 66785295 | G | A | UTR_5_PRIME |  | | SCRN1_BOVIN | | |
| **4** | 71178592 | A | C | NON_SYNONYMOUS_CODING | K549N | | C7orf31 | | |
| **4** | 104385743 | T | G | SYNONYMOUS_CODING | L350 | | JHDM1D | | |
| **4** | 106291332 | G | A | NON_SYNONYMOUS_CODING | R527H | | ENSBTAG00000046616 | | |
| **4** | 107069118 | G | A | DOWNSTREAM |  | | C7orf34 | | |
|  |  | G | A | SYNONYMOUS_CODING | S704 | | KEL | | |
| **4** | 108083522 | C | T | NON_SYNONYMOUS_CODING | A263V | | OR2A12 | | |
| **4** | 108113485 | C | A | SYNONYMOUS_CODING | L26 | | F1MGH4_BOVIN | | |
| **4** | 108114074 | G | A | NON_SYNONYMOUS_CODING | A223T | | F1MGH4_BOVIN | | |
| **5** | 100678553 | G | A | SYNONYMOUS_CODING | L58 | | A5PJG8_BOVIN | | |
| **5** | 100746582 | A | G | NON_SYNONYMOUS_CODING | S178G | | Q95MQ1_BOVIN | | |
| **5** | 101346546 | T | TC | UTR_3_PRIME |  | | A2MG_BOVIN | | |
| **6** | 17043456 | A | T | NON_SYNONYMOUS_CODING | Y182N | | SEC24B | | |
| **7** | 45773867 | C | T | SYNONYMOUS_CODING | P423 | | REXO1 | | |
| **9** | 8455809 | T | G | UTR_3_PRIME |  | | BAI3 | | |
| **9** | 8455809 | T | G | UTR_3_PRIME |  | | BAI3 | | |
| **9** | 13515625 | C | T | SYNONYMOUS_CODING | N799 | | CD109 | | |
| **9** | 23061783 | G | C | UTR_3_PRIME |  | | UB2CB_BOVIN | | |
| **9** | 26448798 | A | G | SYNONYMOUS_CODING | Y183 | | RNF217 | | |
| **9** | 26484818 | G | A | NON_SYNONYMOUS_CODING | T26I | | RNF217 | | |
| **9** | 29703592 | C | A | EXON |  | | ENSBTAG00000046068 | | |
| **9** | 41752281 | A | G | SYNONYMOUS_CODING | S443 | | ARMC2 | | |
| **10** | 45431753 | A | G | UTR_3_PRIME |  | | A6QQI3_BOVIN | | |
| **10** | 45881596 | C | T | DOWNSTREAM |  | | PPIB_BOVIN | | |
|  |  | C | T | SYNONYMOUS_CODING | P160 | | A4FV17_BOVIN | | |
| **10** | 52648893 | G | A | NON_SYNONYMOUS_CODING | L328F | | GRL1A_BOVIN | | |
|  |  | G | A | NON_SYNONYMOUS_CODING | A327V | | GRL1A_BOVIN | | |
| **10** | 69283385 | C | T | NON_SYNONYMOUS_CODING | L156F | | C10H14orf101 | | |
| **10** | 69311034 | G | A | NON_SYNONYMOUS_CODING | S570N | | C10H14orf101 | | |
| **10** | 69314380 | A | T | INTRON |  | | C10H14orf101 | | |
| **10** | 69318649 | G | A | UTR_3_PRIME |  | | C10H14orf101 | | |
| **10** | 70444694 | G | A | EXON |  | | 5S_rRNA | | |
| **11** | 63416473 | C | T | SYNONYMOUS_CODING | S583 | | CEP68 | | |
| **11** | 86246504 | C | A | NON_SYNONYMOUS_CODING | G353C | | GREB1 | | |
| **11** | 93797681 | C | T | SYNONYMOUS_CODING | N62 | | OR1L1 | | |
| **11** | 98408743 | G | T | DOWNSTREAM |  | | C9orf117 | | |
|  |  | G | T | NON_SYNONYMOUS_CODING | L11M | | PTRH1 | | |
|  |  | G | T | UPSTREAM |  | | A6QQZ4_BOVIN | | |
| **11** | 98478308 | C | A | DOWNSTREAM |  | | ENSBTAG00000047785 | | |
|  |  | C | A | SYNONYMOUS_CODING | A361 | | CDK9_BOVIN | | |
| **11** | 98478429 | C | A | DOWNSTREAM |  | | ENSBTAG00000047785 | | |
|  |  | C | A | UTR_3_PRIME |  | | CDK9_BOVIN | | |
| **11** | 98478518 | G | A | DOWNSTREAM |  | | ENSBTAG00000047785 | | |
|  |  | G | A | UTR_3_PRIME |  | | CDK9_BOVIN | | |
| **11** | 98518242 | G | A | DOWNSTREAM |  | | ENSBTAG00000046303 | | |
|  |  | G | A | INTRON |  | | Q1RMV1_BOVIN | | |
| **11** | 105918848 | C | T | DOWNSTREAM |  | | ENSBTAG00000046416 | | |
|  |  | C | T | DOWNSTREAM |  | | GRIN1 | | |
|  |  | C | T | SYNONYMOUS_CODING | C34 | | TMEM210 | | |
| **11** | 105918848 | C | T | UPSTREAM |  | | LRRC26 | | |
| **11** | 106219757 | G | A | SYNONYMOUS_CODING | T1626 | | ABCA2 | | |
| **11** | 106222451 | G | A | INTRON |  | | ABCA2 | | |
|  |  | G | A | SPLICE_SITE_REGION |  | | ABCA2 | | |
| **11** | 106233076 | G | T | UPSTREAM |  | | A6QQL6_BOVIN | | |
|  |  | G | T | UTR_3_PRIME |  | | A6QL90_BOVIN | | |
| **11** | 106391313 | C | T | NON_SYNONYMOUS_CODING | S133N | | LCN8 | | |
|  |  | C | T | UPSTREAM |  | | ENSBTAG00000047297 | | |
| **11** | 106399297 | C | T | UTR_5_PRIME |  | | F1MIA9_BOVIN | | |
| **11** | 106399311 | G | GC | INTERGENIC |  | |  | | |
|  |  | G | GC | UPSTREAM |  | | F1MIA9_BOVIN | | |
| **12** | 15275936 | C | T | EXON |  | | ENSBTAG00000045689 | | |
| **12** | 16097538 | A | T | NON_SYNONYMOUS_CODING | F910I | | ZC3H13 | | |
| **12** | 16119523 | T | C | SYNONYMOUS_CODING | Q323 | | ZC3H13 | | |
| **12** | 28638700 | G | A | DOWNSTREAM |  | | N42L1_BOVIN | | |
|  |  | G | A | NON_SYNONYMOUS_CODING | P3160L | | BRCA2 | | |
|  |  | G | A | SPLICE_SITE_REGION |  | | BRCA2 | | |
| **12** | 28647150 | T | C | NON_SYNONYMOUS_CODING | K3003R | | BRCA2 | | |
| **12** | 34143572 | C | G | UTR_3_PRIME |  | | A5D7B3_BOVIN | | |
| **12** | 36579307 | T | C | SYNONYMOUS_CODING | N461 | | PSPC1_BOVIN | | |
| **12** | 36754405 | A | G | SYNONYMOUS_CODING | L604 | | CENPJ | | |
| **12** | 75682836 | A | G | NON_SYNONYMOUS_CODING | N126D | | ENSBTAG00000039714 | | |
| **13** | 66892584 | A | G | EXON |  | | ENSBTAG00000022562 | | |
| **15** | 18149696 | G | A | SYNONYMOUS_CODING | Q699 | | ATM | | |
| **15** | 18428703 | C | T | NON_SYNONYMOUS_CODING | C145Y | | KDELC2 | | |
| **15** | 43476412 | C | T | INTRON |  | | A6QLJ5_BOVIN | | |
|  |  | C | T | SPLICE_SITE_REGION |  | | A6QLJ5_BOVIN | | |
| **15** | 43476412 | C | T | SPLICE_SITE_REGION |  | | A6QLJ5_BOVIN | | |
| **15** | 43512384 | C | G | SYNONYMOUS_CODING | A480 | | A6H709_BOVIN | | |
| **15** | 44996854 | C | T | NON_SYNONYMOUS_CODING | P63S | | RIC3 | | |
| **15** | 45034439 | G | A | SYNONYMOUS_CODING | P432 | | TUB | | |
| **15** | 48288235 | G | A | UTR_3_PRIME |  | | ENSBTAG00000047088 | | |
| **15** | 48659391 | C | T | NON_SYNONYMOUS_CODING | P158L | | ENSBTAG00000046489 | | |
| **15** | 48707878 | G | C | NON_SYNONYMOUS_CODING | S287T | | UBQLN3 | | |
| **15** | 48707995 | C | A | NON_SYNONYMOUS_CODING | S326Y | | UBQLN3 | | |
| **15** | 48708702 | C | G | NON_SYNONYMOUS_CODING | P562A | | UBQLN3 | | |
| **15** | 54320268 | T | C | NON_SYNONYMOUS_CODING | M544V | | LOC768315 | | |
| **15** | 64105570 | T | C | INTRON |  | | EIF3M | | |
|  |  | T | C | SPLICE_SITE_REGION |  | | EIF3M | | |
| **15** | 64122689 | G | A | NON_SYNONYMOUS_CODING | T499I | | CCDC73 | | |
| **15** | 66755058 | A | T | UTR_3_PRIME |  | | F1MUJ4_BOVIN | | |
| **16** | 29067902 | A | G | NON_SYNONYMOUS_CODING | Q594R | | DNAH14 | | |
| **16** | 55558238 | C | T | UTR_3_PRIME |  | | CYB5R1 | | |
| **16** | 59520698 | G | A | NON_SYNONYMOUS_CODING | T816M | | ASTN1 | | |
| **16** | 62254617 | G | A | SYNONYMOUS_CODING | V127 | | TDRD5 | | |
| **16** | 65628761 | C | T | SYNONYMOUS_CODING | N288 | | LAMC1 | | |
| **16** | 74993031 | A | T | DOWNSTREAM |  | | SERTAD4 | | |
|  |  | A | T | INTERGENIC |  | |  | | |
| **18** | 7823415 | C | T | SPLICE_SITE_REGION |  | | ENSBTAG00000012693 | | |
|  |  | C | T | SYNONYMOUS_CODING | A1951 | | ENSBTAG00000012693 | | |
| **18** | 13749688 | G | A | SYNONYMOUS_CODING | P2431 | | ZNF469 | | |
| **18** | 14593455 | T | C | INTRON |  | | Q32KR9_BOVIN | | |
|  |  | T | C | UTR_3_PRIME |  | | Q32KR9_BOVIN | | |
| **18** | 25912761 | C | A | SYNONYMOUS_CODING | T507 | | CNGB1 | | |
|  |  | C | A | SYNONYMOUS_CODING | T516 | | CNGB1 | | |
|  |  | C | A | SYNONYMOUS_CODING | T526 | | CNGB1 | | |
|  |  | C | A | SYNONYMOUS_CODING | T530 | | CNGB1 | | |
| **18** | 25912761 | C | A | SYNONYMOUS_CODING | T972 | | CNGB1 | | |
|  |  | C | A | SYNONYMOUS_CODING | V304 | | CNGB1 | | |
|  |  | C | A | SYNONYMOUS_CODING | V313 | | CNGB1 | | |
|  |  | C | A | SYNONYMOUS_CODING | V323 | | CNGB1 | | |
|  |  | C | A | SYNONYMOUS_CODING | V327 | | CNGB1 | | |
|  |  | C | A | SYNONYMOUS_CODING | V769 | | CNGB1 | | |
| **18** | 46249680 | G | A | DOWNSTREAM |  | | FFAR1 | | |
|  |  | G | A | NON_SYNONYMOUS_CODING | E312K | | B9VJW0_BOVIN | | |
|  |  | G | A | NON_SYNONYMOUS_CODING | E312K | | B9VJW0_BOVIN | | |
| **18** | 48630635 | G | C | DOWNSTREAM |  | | Q0V8L0_BOVIN | | |
|  |  | G | C | DOWNSTREAM |  | | Q0V8L0_BOVIN | | |
|  |  | G | C | SYNONYMOUS_CODING | L4997 | | RYR1 | | |
| **18** | 48853763 | T | A | NON_SYNONYMOUS_CODING | E240V | | RINL | | |
|  |  | G | A | DOWNSTREAM |  | | ERCC1_BOVIN | | |
|  |  | G | A | UPSTREAM |  | | Q0VCZ5_BOVIN | | |
| **18** | 53451585 | G | A | UTR_3_PRIME |  | | Q0VD10_BOVIN | | |
|  |  | C | T | UTR_3_PRIME |  | | PNML1_BOVIN | | |
| **19** | 5384204 | G | A | INTRON |  | | ENSBTAG00000016078 | | |
|  |  | G | A | SPLICE_SITE_REGION |  | | ENSBTAG00000016078 | | |
| **19** | 23441452 | C | T | SYNONYMOUS_CODING | A661 | | SMYD4 | | |
| **19** | 27702231 | C | T | DOWNSTREAM |  | | Q1RMW0_BOVIN | | |
|  |  | C | T | SYNONYMOUS_CODING | G320 | | E1BD38_BOVIN | | |
| **20** | 33405113 | G | A | SYNONYMOUS_CODING | A904 | | C6 | | |
| **20** | 33405337 | C | T | UTR_3_PRIME |  | | C6 | | |
| **20** | 33405464 | A | G | UTR_3_PRIME |  | | C6 | | |
| **20** | 33550404 | T | A | UTR_3_PRIME |  | | C7 | | |
| **20** | 38040315 | C | T | SPLICE_SITE_REGION |  | | NADKD1 | | |
| **20** | 38040315 | C | T | SYNONYMOUS_CODING | L101 | | NADKD1 | | |
| **20** | 60955375 | T | C | EXON |  | | ENSBTAG00000047588 | | |
| **20** | 62630546 | C | T | START_GAINED |  | | DAP1_BOVIN | | |
|  |  | C | T | UTR_5_PRIME |  | | DAP1_BOVIN | | |
| **20** | 62695696 | C | T | UTR_3_PRIME |  | | DAP1_BOVIN | | |
| **20** | 62759018 | G | A | START_GAINED |  | | ANKRD33B | | |
|  |  | G | A | SYNONYMOUS_CODING | H131 | | ANKRD33B | | |
|  |  | G | A | UTR_5_PRIME |  | | ANKRD33B | | |
| **21** | 44702435 | C | A | INTRON |  | | NPAS3 | | |
| **22** | 49232333 | C | G | DOWNSTREAM |  | | A7Z085_BOVIN | | |
|  |  | C | G | NON_SYNONYMOUS_CODING | A509G | | Q56GY1_BOVIN | | |
| **22** | 50887724 | G | C | NON_SYNONYMOUS_CODING | A255G | | RBM6 | | |
| **23** | 27101468 | G | A | NON_SYNONYMOUS_CODING | P2414S | | TNXB | | |
| **23** | 28304727 | G | A | NON_SYNONYMOUS_CODING | E131K | | F1MGC3_BOVIN | | |
|  |  | G | A | INTRON |  | | F1MWX8_BOVIN | | |
|  |  | G | A | SPLICE_SITE_REGION |  | | F1MWX8_BOVIN | | |
| **23** | 28537300 | C | T | NON_SYNONYMOUS_CODING | R235Q | | ENSBTAG00000037421 | | |
| **23** | 28537343 | C | A | NON_SYNONYMOUS_CODING | G221W | | ENSBTAG00000037421 | | |
| **23** | 28540016 | CAGA | C | CODON_CHANGE_PLUS_CODON_DELETION | FC35C | | ENSBTAG00000037421 | | |
| **23** | 29149992 | G | C | NON_SYNONYMOUS_CODING | V19L | | OR12D3 | | |
| **23** | 30135859 | C | T | NON_SYNONYMOUS_CODING | R315Q | | PGBD1 | | |
| **23** | 30153145 | G | A | SYNONYMOUS_CODING | S158 | | PGBD1 | | |
| **23** | 31646550 | G | C | NON_SYNONYMOUS_CODING | V112L | | E1BK75_BOVIN | | |
|  |  | G | C | UPSTREAM |  | | H31_BOVIN | | |
|  |  | G | C | UPSTREAM |  | | ENSBTAG00000007286 | | |
| **23** | 32839446 | C | T | DOWNSTREAM |  | | A0JNC5_BOVIN | | |
|  |  | C | T | NON_SYNONYMOUS_CODING | V33I | | A6QQ83_BOVIN | | |
| **23** | 32951247 | T | G | UTR_3_PRIME |  | | E1BDP3_BOVIN | | |
| **23** | 32959994 | G | A | SYNONYMOUS_CODING | F324 | | E1BDP3_BOVIN | | |
| **23** | 32967267 | G | A | NON_SYNONYMOUS_CODING | P173L | | E1BDP3_BOVIN | | |
| **23** | 34807599 | T | G | SYNONYMOUS_CODING | A167 | | Q5R1X5_BOVIN | | |
| **23** | 39164505 | A | C | DOWNSTREAM |  | | KDM1B | | |
|  |  | A | C | UTR_3_PRIME |  | | A5PJQ1_BOVIN | | |
| **23** | 39164514 | CAG | C | DOWNSTREAM |  | | KDM1B | | |
|  |  | CAG | C | UTR_3_PRIME |  | | A5PJQ1_BOVIN | | |
| **23** | 39646804 | G | A | UTR_3_PRIME |  | | F1N715_BOVIN | | |
| **23** | 44171707 | C | G | NON_SYNONYMOUS_CODING | A1571P | | HIVEP1 | | |
| **23** | 44171709 | C | A | NON_SYNONYMOUS_CODING | R1570L | | HIVEP1 | | |
| **24** | 30060724 | G | A | EXON |  | | ENSBTAG00000034032 | | |
|  |  | G | A | INTRON |  | | Q1LZ85_BOVIN | | |
| **24** | 32887666 | T | C | INTRON |  | | OSBPL1A | | |
| **24** | 54047364 | C | T | NON_SYNONYMOUS_CODING | V190M | | MBD2 | | |
| **25** | 2867550 | G | T | START_GAINED |  | | NAT15_BOVIN | | |
|  |  | G | T | UTR_5_PRIME |  | | NAT15_BOVIN | | |
| **25** | 2993664 | C | G | DOWNSTREAM |  | | TRAP1_BOVIN | | |
|  |  | C | G | DOWNSTREAM |  | | bta-mir-2383 | | |
|  |  | C | G | STOP_GAINED | Y85* | | DNAS1_BOVIN | | |
|  |  | C | G | STOP_GAINED | Y85* | | DNAS1_BOVIN | | |
| **28** | 25381049 | G | A | NON_SYNONYMOUS_CODING | R205H | | A4FV23_BOVIN | | |
| **28** | 25708392 | G | A | UPSTREAM |  | | A6QP28_BOVIN | | |
| **28** | 25708392 | G | A | UTR_3_PRIME |  | | VP26A_BOVIN | | |
| **28** | 28304946 | C | T | UTR_3_PRIME |  | | A7MB04_BOVIN | | |
| **28** | 33744395 | C | T | SYNONYMOUS_CODING | K870 | | Q29S04_BOVIN | | |
| **28** | 44153950 | C | T | SYNONYMOUS_CODING | D244 | | CHAT | | |
|  |  | G | A | NON_SYNONYMOUS_CODING | R463H | | Q08E65_BOVIN | | |
|  |  | G | A | UTR_3_PRIME |  | | Q08E65_BOVIN | | |
| **28** | 44820219 | C | T | SYNONYMOUS_CODING | H177 | | OR13A1 | | |
| **29** | 12753316 | C | T | UTR_3_PRIME |  | | RAB30_BOVIN | | |
| **29** | 26291033 | A | G | NON_SYNONYMOUS_CODING | K174E | | A6QQD4_BOVIN | | |
| **29** | 50379124 | A | G | SYNONYMOUS_CODING | F220 | | CRLF2 | | |
| **29** | 50384685 | C | T | SYNONYMOUS_CODING | T51 | | CRLF2 | | |
| **X** | 16859873 | C | T | SYNONYMOUS_CODING | K210 | | USP26 | | |
| **X** | 20274126 | A | T | NON_SYNONYMOUS_CODING | L775H | | ARHGEF6 | | |
| **X** | 21969353 | A | G | NON_SYNONYMOUS_CODING | N224S | | Q0II42_BOVIN | | |
| **X** | 83599354 | G | A | NON_SYNONYMOUS_CODING | S551N | | ERC6L_BOVIN | | |
| **X** | 90676723 | G | A | SYNONYMOUS_CODING | G271 | | PHF16 | | |
| **X** | 91754434 | C | T | SYNONYMOUS_CODING | A523 | | TBC1D25 | | |
| **X** | 93637756 | G | C | EXON |  | | U6 | | |
| **X** | 108886418 | C | G | SYNONYMOUS_CODING | V89 | | BCOR | | |
| **X** | 118122262 | G | C | SYNONYMOUS_CODING | P380 | | GLPK_BOVIN | | |
| **X** | 118636994 | C | T | UTR_5_PRIME |  | | Q2T9P7_BOVIN | | |
| **X** | 141157032 | C | T | NON_SYNONYMOUS_CODING | V1050I | | FRMPD4 | | |
