# Supplementary material for: Epidermolysa bullosa in Danish Hereford calves is caused by a deletion in LAMC2 gene
Source: BMC Vet Res. 2015 Feb 7;11:23. doi: 10.1186/s12917-015-0334-8 (PMC4328060; doi:10.1186/s12917-015-0334-8)
Supplement: Additional file 4: — Private larger deletions detected in epidermolysis bullosa case 1. Deletion in encompassing LAMC2 exon 1 is highlighted. [file 12917_2015_334_MOESM4_ESM.docx]

**Additional file 4. Private larger deletions detected in epidermolysis bullosa case 1.** Deletion in encompassing *LAMC2* exon 1 is highlighted. CHR: Chromosome; POS: position; SVLEN: size of the deletion.

| CHR | POS | SVLEN |  | CHR | POS | SVLEN |
| --- | --- | --- | --- | --- | --- | --- |
| 1 | 47278703 | 1865 |  | **12** | 68841745 | 1012 |
| 2 | 2026854 | 2015 |  | **13** | 60300797 | 2028 |
| 2 | 15655531 | 117 |  | **14** | 46620941 | 784 |
| 2 | 38834294 | 1034 |  | **15** | 11856187 | 744 |
| 3 | 89476543 | 2167 |  | **15** | 15747705 | 22611 |
| 4 | 93973491 | 355 |  | **15** | 43924322 | 2815 |
| 4 | 106699434 | 130 |  | **15** | 74438148 | 52480 |
| 4 | 106867355 | 2399 |  | **15** | 75038507 | 2159 |
| 4 | 120107942 | 5066 |  | **15** | 75040810 | 2168 |
| 5 | 9363450 | 1885 |  | **16** | **65704617** | **2433** |
| 5 | 89662718 | 270 |  | **17** | 28242535 | 2136 |
| 6 | 10649120 | 977544 |  | **17** | 71617545 | 11564 |
| 6 | 21224691 | 219368 |  | **18** | 51752340 | 36921 |
| 6 | 39051945 | 1315 |  | **19** | 61279645 | 3380 |
| 6 | 51251929 | 140 |  | **20** | 803901 | 113 |
| 7 | 11714209 | 134 |  | **20** | 35340114 | 578 |
| 7 | 49812166 | 1123 |  | **20** | 59884782 | 135 |
| 8 | 27246784 | 106 |  | **20** | 60935987 | 5582 |
| 8 | 77746873 | 764 |  | **22** | 5664360 | 14656 |
| 8 | 86457510 | 32912 |  | **22** | 46397045 | 77449 |
| 8 | 92885800 | 836 |  | **23** | 31935981 | 1119 |
| 8 | 94109539 | 1969 |  | **26** | 42787425 | 6116 |
| 9 | 40391809 | 4744 |  | **27** | 28160398 | 131 |
| 10 | 31605264 | 772 |  | **28** | 7101977 | 7889 |
| 10 | 31798768 | 1196 |  | **28** | 36456372 | 3380 |
| 11 | 55640213 | 7962 |  | **29** | 49070502 | 378 |
| 12 | 3328348 | 905 |  | **X** | 18814427 | 264304 |
| 12 | 9188213 | 1858 |  | **X** | 29273174 | 185 |
| 12 | 37885138 | 352 |  | **X** | 71885833 | 21953 |
| 12 | 37895112 | 1728 |  | **X** | 141557117 | 935 |
